# High proportions of obstetric referrals in Addis Ababa: the case of term premature rupture of membranes

**DOI:** 10.1186/s13104-016-1852-6

**Published:** 2016-01-25

**Authors:** Alemnesh H. Mirkuzie, Mitike Molla Sisay, Mulu Muleta Bedane

**Affiliations:** Center for International Health, University of Bergen, Årstadv 21, Overlegedanielsenshus, 5020 Bergen, Norway; School of Public Health, College of Health Sciences, Addis Ababa University, Addis Ababa, Ethiopia; WAHA International, University of Gondar, Post Box 41822, Gondar, Ethiopia

**Keywords:** Diagnosis, Health center, Hospital, Management, Mis-diagnosis, Guidelines, PROM, Referral

## Abstract

**Background:**

The Public Health Centers (HCs) provide basic obstetric and neonatal care to about 80 % of the eligible population in Addis Ababa. Hospitals provide comprehensive services and are referral centers for complications that cannot be managed at the HCs. This study assessed the proportion of obstetric referrals in general and referrals due to premature rupture of membranes (PROM) at term in particular, from the HCs in Addis Ababa and explored its appropriateness and management in hospitals.

**Methods:**

The study used a sequential explanatory mixed methods design. Routine retrospective data were collected from ten randomly selected HCs in 2012. Key informant interviews were conducted using a guide developed following a preliminary analysis of the quantitative data. Ten head midwives, one from each health center participated in the interviews.

**Results:**

Of the 9340 mothers who sought skilled birth care in the ten HCs in 2012, 2820 (30.3 %) were diagnosed with obstetric complications and referred to hospital. Term PROM accounted for 557 (19.7 %) of the referrals and it was widely varied across the HCs. Fifteen (7.8 %) mothers who were referred for PROM, had intact membranes upon hospital examinations. Forty-two (77.8 %) of the referred mothers who had spontaneous labour and delivery could have been misclassified as not having labour upon referral. In the interviews, variations in diagnosing and managing term PROM were identified as themes. Three HCs relayed solely on mothers’ self reports of amniotic fluid leakage to diagnose, two HCs did complementary speculum/vaginal examination, three HCs monitored sign of labour on top of confirming the leakage. Regarding management, two HCs practiced expectant management, three referred mothers after 30 min of observation while others issued referral right away. All providers reported the lack of clinical guidelines for most common obstetric problems in their HC.

**Conclusions:**

The study reported large proportion of obstetric referrals in general and PROM referrals in particular as well as variations in diagnosing and managing term PROM. These could largely be attributed to lack of clinical guidelines for most common obstetric complications at the HCs and competency gap among providers. Addressing the identified gaps and strengthening the primary care settings could contribute to improved quality of obstetric care and outcomes.

**Electronic supplementary material:**

The online version of this article (doi:10.1186/s13104-016-1852-6) contains supplementary material, which is available to authorized users.

## Background

Premature rapture of membranes (PROM) is a condition when amniotic membranes break before the onset of labour [1, 2]. PROM can occur before the pregnancy reaches to term or at term. When PROM occurs at ≤37 weeks of gestation, it is called pre-term PROM. When PROM occurs at 37 completed weeks of gestation or after it is called term PROM [1]. Pre-term PROM is often associated with preterm birth and an increased risk of neonatal morbidity and mortality [2]. This paper focuses on term PROM that occurs in about 10 % of the pregnancies. In 85 % of term PROM cases, labour starts spontaneously within 24 h [2, 3]. The best way to diagnose PROM is observing amniotic fluid leakage in a speculum examination while ferning (visualization of fern like structure under a microscopic examination of vaginal swab) and Nitrazine test can also be used [4]. The course of labour might be complicated if PROM is accompanied by cord prolapse, which could endanger the life of the unborn child. Prophylactic antibiotics are indicated after 18 h (in prolonged PROM) for the prevention of chorioamnionitis [1, 5, 6].

Clinical guidelines (protocols) are tools developed to facilitate diagnosis, management and treatment of clinical conditions. Guidelines incorporate cutting-edge evidence for improving quality of care and patient outcomes. They remove variations in clinical practices, can support quality assurance and can also provide mechanisms to hold professionals accountable for their practices [7, 8]. The guidelines can be developed at national, regional and local levels. In 2011, the Federal Ministry of Health of Ethiopia has developed national management guidelines for common obstetric complications, including PROM for tertiary care level (hospitals) [9]. Whereas management guidelines are still unavailable for most common obstetric complications, including PROM at the primary Health Centers (HCs) where the majority of mothers and children receive care.

In Ethiopian, the public primary HCs provide basic obstetric and neonatal care (EmONC) and are expected to mange about 85 % of the total deliveries. Hospitals are a tertiary care centers for referrals and provide comprehensive EmONC for about 15 % complications that cannot be managed at the primary HCs. These two health care settings are linked by a system of referral. Referrals could facilitate prompt access to emergency care for mothers with obstetric complications and has the potential to reduce adverse maternal and neonatal outcomes [10]. Contrary to what is expected, 65 % of the total deliveries in Addis Ababa occur in hospitals with the majority being referred during the intra-partum period. These undue referrals seem overburdening the hospitals and contributing to the compromised quality of obstetric and neonatal care in the city [11, 12].

Poor competence among skilled providers, lack of clinical management protocols at the primary HCs and poor adherence to clinical recommendations among providers could have a major contribution to the high burden of referrals and to the poor maternal and neonatal health outcomes [10, 11]. In this regard Ethiopia is not exceptional. Several studies from other sub-Saharan countries have shown similar patterns of over-diagnosing obstetric complications at the primary care settings where the magnitude of referrals reaching as high as 43 % in Zimbabwe (30 % antenatal and 13 % intrapartum) [13–15]. Studies also show that by strengthening the primary care settings much of the undue referrals could have been averted without compromising maternal and neonatal wellbeing [10, 16].

While working with primary care providers in Addis Ababa, the high burden of term PROM and the widespread knowledge gaps on its diagnosis and management was a concern for our research team. We believe that the evidence generated in this study could have paramount importance in informing local practices and for generating knowledge that could be transferred to other similar settings and contexts. The study assessed the proportion of obstetric referrals in general and referrals due to term PROM in particular from the primary HCs to hospitals and outcomes of labour at the hospitals among the referred cases. The study also explored how skilled providers in the HCs diagnosis and mange term PROM and the major gaps impacting their day-to-day obstetric practice.

## Methods

### Study settings

The study was conducted in Addis Ababa, the capital of Ethiopia as part of an intervention project that intends to improve maternal and neonatal health outcomes through intensive knowledge and skills training for midwives/nurses on basic EmONC. The city is home for about 3.5 million people and is administratively divided in 10 sub-cities. Under the City Administration, Health Bureau, there are over 90 public primary HCs, which provide basic EmONC and four regional public hospitals providing comprehensive EmONC. These hospitals are Zewditu Memorial, Ghandi Memorial, Tirunesh Beijing and Yekatit 12. Ghandi Memorial is a maternity hospital while the other three are general hospitals that receive referrals from all over the city. Moreover, there are federal specialized referral hospitals in the city, which also provide comprehensive EmONC. Generally speaking availability, accessibility and acceptability of EmONC services are quite high in the city [11, 12]. The numbers of basic and comprehensive EmONC facilities outnumber the WHO minimum standard and the median distance to the nearest comprehensive EmONC facility is 5 km. Eight five percent of mothers in the city give birth in health facilities with the great majorities at public facilities.

### Study design and sample selection

Using a mixed methods approach, this study collected quantitative and qualitative data. The study employed a sequential explanatory design. Routine retrospective data were collected from registers and preliminary analyses were made. This was followed by qualitative interviews to explore the issues behind the numbers and come up with plausible explanations.

The study used Consolidated Criteria for Reporting Qualitative Research (COREQ) guideline for the qualitative findings [17] (see Additional file 1).

To have a representative sample, ten well-established primary public HCs were randomly selected one from each sub-city using a lottery system. All the selected HCs are primary care facilities providing basic EmONC and have a similar staffing profile. The HCs provide delivery services free of charge and serve heterogeneous, low-income group of women as many women from high-income strata opting for private facilities. Hospital data were collected from two regional and one federal referral hospitals, which all together attend about two-thirds of the deliveries in the public health facilities in Addis Ababa. Zewditu Memorial and Ghandi Memorial hospitals were selected randomly from the four hospitals under the Addis Ababa City Administration Health Bureau. The third hospital was Tikur Anbessa, a federal referral hospital randomly selected from the two federal referral hospitals, catering obstetric and neonatal care services in Addis Ababa.

### Quantitative data collection and analysis

This review retrieved retrospective data from labour, delivery and intrapartum referral logbooks from the selected HCs. These included total number of women who sought care during labour and delivery, number of total referrals, referrals due to term PROM and number of full-time skilled providers from January 1st, 2012 to December 30, 2012.

To identify women who were referred with a diagnosis of PROM, routine hospital referral registers were first checked. By using their unique patient identifiers, in total 227 individual patient records were retrieved and reviewed. Women referred for other obstetric and neonatal complications were not included. The hospital data were collected using checklists to assess the standards of care for term PROM and for assessing maternal and neonatal outcomes. The checklist included maternal age, gravidity, parity, last menstrual period, gestational age, referring facility, diagnosis made by the referring primary health center, diagnosis after hospital arrival, prophylactic antibiotic, time referred from the referring facility, arrival time in hospital, admission to hospital, if a mother was not admitted to the hospital and referred again, reason for second or third referral from a hospital, time of delivery/induction or caesarean section, action at the hospital (induction, augmentation or caesarean section) and mode of delivery. Mean and range values were calculated for continuous data, while proportions and Chi square tests were calculated for categorical data.

### Definitions of terms and synonyms

In this study a mother is said to have term PROM when she fulfils the following three criteria [1, 2]: (1) she should be at 37 completed weeks of gestation or more, (2) the amniotic membranes should have ruptured, (3) she should not be in labour. Due to the difficulty to ascertain whether the woman was in labour or not upon referral, we used the time taken from referral to delivery as a proxy indicator. By definition normal labour could take 12 h in multigravida and 18 h in primigravida mothers [18, 19]. Taking into consideration the travel time and logistic challenges to reach from HCs to hospitals or from hospitals to hospitals, we set 9 h as a cutoff. Therefore, a mother who had spontaneous labour and vaginal delivery within 9 h of referrals were considered to be in labour by the time the referral was issued and the rupture of amniotic membranes for this mother could have been a sign of labour. Those women who had ruptured amniotic membranes and who had spontaneous labour and delivery after 9 h of referral were considered to have PROM.

In this study primary HCs are also referred as basic EmONC facilities or public HCs. Tertiary hospitals are also referred as comprehensive EmONC facilities or hospitals.

### Qualitative interviews

Key informant interviews were conducted. Ten head midwives, one from each HC were approached for the interviews and all of them agreed to participate. Using a focused interview guide, the interviews were conducted after obtaining informed verbal consent. The guide explored experiences on (1) how PROM diagnosis was made? and (2) how mothers with term PROM were managed at health center? (see Additional file 2). All the interviews were conducted in Amharic, the national language fluently spoken by all the interviewees and the interviewer. The principal investigator (PI) did all the interviews in the HCs. During the interviews, dialogues were made to continue to the point where no new information was coming up and took on average 15 min. Notes taken during the interviews were transcribed and then translated to English for analyses by the PI. Doing the transcriptions and translations allowed the PI to get immersed into the data for gaining an overall impression of the findings. The interviews were analysed using content analysis. According to the principles in content analysis [20–22], the interview transcripts were first read and re-read to have an overview of the data. Then by using the two major questions from the interview guide, two themes were identified. The first theme was ‘making PROM diagnosis’ and the second theme was ‘managing PROM’. Then after the interview transcripts were sorted out and aligned with the respective themes, which then followed quantitative interpretations of the findings as shown in Table 1. In qualitative content analysis, the process of data analysis also involves interpretation of findings and can be presented in the form of frequency [20–22]. To ensure the validity of the findings, we did participant checking by calling up the informants in a consultative meeting where we presented preliminary findings. At the meeting, the informants gave us positive feedbacks that the findings presented were the main issues addressed during the in-depth interviews.

**Table 1:** Shows variable diagnostic and management approaches for term PROM explored in key informant interviews in Addis Ababa

Diagnosis	Management	
	Immediate referral to hospital	Observe labour progress for eight hours in the health center
1. Mother’s self-report of leakage	Three HCs	
2. Mother’s self-report of leakage + observing signs of true labour for 10 min	Two HCs	
3. Mother’s self-report of leakage + speculum/vaginal exam + observing signs of true labour for 30 min	Three HCs	Two HCs

### Ethical considerations and study permits

The project obtained ethical approval from the Ethics Committee of Addis Ababa City Administration, Health Bureau (AACAHB), and the Ethics Committee in Western Norway. Study permits were obtained from the AACAHB, sub-city health bureaus, hospitals and health centers. First, the AACAHB issued us a support letter for all sub-cities health bureaus. Based on the support letter, we got permission from each sub-city health bureau to access all the health facilities under them. Finally, in reference to the support letter from the sub-cities, the head of each health facility granted us access to the registers and to do the qualitative interviews. As stated in the protocol approved by the ethics committees’, informed verbal consent to participate in the in-depth interviews was secured from each informant after explaining the purpose of the study. Prior to the interviews all the informants were informed about the interview procedure and their right to opt out at any point during the interviews without any consequence. For ethical reasons, instead of their names all the HCs were de-identified using capital letters.

## Results

### Quantitative findings

#### Referrals and PROM referrals in primary HCs

A total of 9340 pregnant women had visited the study HCs for labour and delivery care in 2012. Of which, 6520 women gave birth at the HCs while 2820 (30.3 %) were identified to have complications requiring referral to hospitals for management and care. The proportions of obstetric referrals widely varied from 18.3 to 51.3 %. Term PROM accounted for 557 (19.7 %) of the referrals with wide variations across the 10 HCs ranging between 6.7 and 45.1 % (Table 2).

**Table 2:** Mothers who sought care in HCs during labour and delivery, referrals to hospitals for obstetric complications and for term PROM and skilled providers for the caseloads at each HC in 2012, Addis Ababa

HC	Number of mothers who sought care during labour	Number (proportion) of overall obstetric referrals	Number (proportion) of PROM referrals	Number of full time skilled providers actual (expected)^a^
A	733	173 (23.6 %)	78 (45.1 %)	6 (3.4)
B	570	234 (41.1 %)	60 (25.6 %)	6 (2.1)
C	564	184 (32.6 %)	28 (15.2 %)	8 (2.3)
D	651	248 (38.1 %)	69 (27.8 %)	5 (2.4)
E	1752	493 (28.1 %)	117 (23.7 %)	5 (7.6)
F	1261	365 (28.9 %)	44 (12.1 %)	10 (5.4)
G	730	187 (25.6 %)	27 (14.4 %)	9 (3.3)
H	1770	457 (25.8 %)	98 (21.4 %)	12 (7.9)
I	725	372 (51.3 %)	25 (6.7 %)	6 (2.1)
J	584	107 (18.3 %)	11 (10.1 %)	5 (2.9)
Total	9340	2828 (30.3 %)	557 (19.7 %)	72 (39)

^a^Expected number of skilled providers for the case loads were calculated based on six skilled providers for 1000 births per year, a standard set in the State of the World’s Midwifery, 2011 report

Number of full time skilled providers in the HCs varied between five and 12. HC H had the highest number of deliveries attended as well as the highest number of providers. Table 2 also showed the required number of providers for the number of deliveries each HC attended in 2012. For calculating the acceptable ratio of skilled providers for the attended deliveries, we used the 2011 State of the World´s Midwifery recommendation of six skilled providers for 1000 deliveries [23]. Based on this recommendation, in 2012, HC E had less skilled providers for its caseload, while the rest had excess skilled providers for their caseload. All the HCs reporting high proportions of referrals and term PROM referrals had excess skilled providers for their caseload (Table 2).

#### Hospital management and delivery outcomes

According to data from the hospitals, in Ghandi Memorial the main maternity referral hospital in the city, PROM accounted for 26 % of all obstetric referrals. Of the 227 PROM cases reviewed in the three hospitals, the average age of the mothers was 26 years, 127 (60 %) were primigravida, 166 (82.2 %) knew their last menstrual period and 191 (84.1 %) had term pregnancy. The majorities i.e. 104 (56.2 %) of the referrals were from the HCs (Table 3).

**Table 3:** Characteristics of mothers referred to hospitals with PROM diagnosis in 2012, Addis Ababa

Variable	Value
Age	
Range	17–38 years
Mean	26 year
Mode	25 year
Number of pregnancy	
Primigravida	127 (60 %)
2 or more pregnancies	100 (40 %)
Last menstrual period	
Known	166 (82.2 %)
Unknown	36 (16 %)
Gestational age	
Term	191 (84.1 %)
Pre-term	36 (15.9 %)
Referred from	
Public HCs	104 (56.2 %)
Public hospitals	72 (38.9 %)
Private facilities	9 (4.9 %)

NB: Due to missing data the numbers may vary and may not add up to the total 227

Of the 173 mothers with term pregnancy and having ruptured amniotic membrane, 148 (85.5 %) received prophylactic antibiotics and Ampicillin was the drug of choice. Hospital admissions were issued in 124 (71.7 %) of the mothers. The 49 (28.3 %) mothers who did not get admission were referred to other hospitals. Major reasons for the referrals from one hospital to another were shortage of obstetric beds and unavailability of senior obstetricians. Among the 124 mothers who got hospital admission, 70 (60 %) had spontaneous labour and 52 (40 %) required induction to start up labour or augmentation to speed up labour. In total, 73 (59.3 %) had an unassisted vaginal delivery, 32 (24 %) had cesarean delivery and 18 (14.6 %) had forceps assisted vaginal delivery.

#### Travel time and duration of labour

The average time the referred mothers had taken to reach to the referral hospitals was 1.5 h. The time from hospital admission to spontaneous vaginal delivery ranged from 30 min to 48 h. Thirty-two (59.3 %) of the mothers who had spontaneous labour and vaginal birth had delivered within 6 h of hospital admission and 42 (77.8 %) within 9 h.

#### Misdiagnosis of term PROM

Of 191 referred mothers having had term pregnancies, 15 (7.8 %) were found to have intact amniotic membranes during physical examinations at the hospital with no significant difference between primigravida and multigravida mothers (p > 0.05). Fifty-four mothers with ruptured amniotic membranes at presentation to the hospital had spontaneous labour and vaginal delivery. Of which 42 (77.8 %) delivered within nine hours of hospital admission. As per the given operational definition, these mothers could have been in labour when they were issued the referrals.

#### Qualitative findings

All the 10 key informants interviewed expressed their concern about the lack of practice guidelines for diagnosing and managing term PROM. All the interviewed midwives could differentiate pre-term PROM from term PROM when it comes to diagnostic approaches. Whilst, six of the ten interviewed providers did not know the different management approaches for pre-term and term PROM.

According to the interviews, PROM diagnosis was made solely based on a mother’s self report of amniotic fluid leakage in three HCs. HC A, B and C mothers who reported having amniotic fluid leakage were considered as PROM without making any confirmatory examinations and were referred to hospitals right away. “if a mother said that her water is broken, we consider her as PROM and we will immediately refer her to hospital for management” (an informant from HC A) (Table 1).

In HC D and E, mothers who reported leakage of fluid were monitored for uterine contraction for 10 min to rule out whether they were in labour or not. Mothers with no uterine contraction in 10 min were considered to have term PROM and were referred to the hospital right away. According to the interviews with the head midwives in these HCs, term PROM was associated with a greater risk of complications that they would not have the skills and the resources to handle. According to the midwives in these HCs, cord prolapse and endometritis are eminent for mothers with PROM whereby and the providers expressed their insecurity about their skills to manage such cases. “… managing mothers with term PROM is beyond our scope of midwifery practice …we do not let mothers with term PROM stay in our health center” (an informant from HC D). The midwives in these HCs had also expressed their concern that they had large proportions of mothers’ diagnosed with PROM in their HCs (Table 1).

HC F, G and H were diagnosing term PROM by observing leakage of amniotic fluid by speculum/vaginal examinations and observing the mothers for 30 min to check for sign of labour. Mothers with no uterine contraction and with confirmed amniotic fluid leakage were considered to have term PROM and were immediately referred to hospital for labour and delivery management. “When a mother comes to our health center with a complaint of leakage of amniotic fluid, we do speculum and digital vaginal examination to make sure that the amniotic membrane has actually ruptured” (an informant from HC H). Mothers with contraction and confirmed leakage of amniotic fluid were considered to have labour associated rupture of membrane, a normal physiologic change that happens during labour (Table 1).

In HC I and J, diagnosis of PROM were made the same way as in HC F, G and H by observing leakage in speculum/vaginal examination and observing uterine contraction for at least 30 min. The difference between these two groups of HCs was the management approach. In HC I and J, mothers with PROM could be observed for about 8 h before the referral. According to the providers in HC F, H, I and J they could manage term PROM in their health centers when maternal and foetal conditions were reassuring (Table 1). “… when we get mothers saying that their water has broken, after we evaluate them, they will be admitted to our health center and observed for about eight hours. If there is no spontaneous labour in eight hours we refer them to hospital after giving them a loading dose of Ampicillin… in the referral slip we write how long the mothers had been observed in our health center” (an informant from HC I).

## Discussion

The findings have shown that about a third of pregnant mothers seeking care at the HCs in Addis Ababa were referred to hospitals and 20 % of the referrals were due to term PROM. Hospital records revealed that the great majorities of the referred women as cases of term PROM could have been misdiagnosed due to failure in the part of the providers to ascertain conditions of amniotic membranes and signs of labour. According to the qualitative accounts, there seem to be widespread misunderstanding among the providers what constitutes PROM, how to diagnose it and how to manage leading to variations in making PROM diagnosis and management approaches. HC relying solely on mothers self reports of leakage to diagnose PROM recorded the highest proportions of PROM referrals and the highest burden of misdiagnosis. The most effective management approach in terms of reducing referrals was expectant management in the HC for at least 8 h. Poor competency among providers and lack of standard PROM management guidelines in the HCs might have implications for the observed over-diagnosis and misdiagnoses as well as for the variable diagnostic and management practices across the primary heath centers.

The large proportions of the misdiagnosis happened due to misclassification. For one 7.8 % of the mothers with intact membranes were misclassified in the HCs as having intact membranes. For the other, the great majority of mothers presented with leakage were most likely in labour when they presented in the HCs but they were misclassified as not having labour. The hospital data revealed that about 60 % and about 80 % of the mothers who were referred to hospitals had spontaneous vaginal delivery within 6 and 9 h respectively. Knowing the fact that the average duration of labour in primigravida lasts 18 h and 12 h in multigravida [18, 19], over three quarters of the mothers referred as PROM were actually in labour when they were referred and the leakage of amniotic fluid could be the normal physiology that happens during labour.

The short time the health care providers took to check on the onset of labour and the fact that they did not perform a speculum/vaginal examinations to rule out whether the membranes have ruptured or not could also have significant contribution to the misclassifications. Consistent with our findings, in a recent study from Tanzania it was reported that not giving enough time for monitoring mothers with obstetric conditions have had negative implications on obstetric management and outcomes [15]. Speculum examination could help to visualize pooling of amniotic fluid, which could give reliable evidence when complemented with self-report of leakage. Moreover, it could possibly help to check the cardinal signs of true labour, including effacement and dilatation of the cervix [1]. Nevertheless, in a report from Atlanta, the United States of America, Nurse Midwives expressed their concerns that using speculum examination in the case of PROM does not provide sufficient information on labor progress unless it is complemented by a digital vaginal examination [1].

HCs making proper PROM diagnosis and offering expectant management for eight hours reported acceptable proportions of PROM referrals ranging from 7 to 12 %. Expectant management remains an option for mothers with term PROM since the majority would have spontaneous birth within 24 h. Midwifery management of term PROM in two hospitals in the USA is to defer induction and to await spontaneous labour for 12–18 h [1]. In line with international recommendations, hospital management of term PROM in Ethiopian includes expectant management followed by induction of labour between 12 and 16 h of membranes rupture [1, 9, 24]. In the absence of co-morbidities and when maternal and fetal conditions are reassuring, considering the primary care settings to undertake the expectant management for at least 8–10 h of membranes rupture could have several benefits including; (1) it could significantly reduce referrals to hospitals as the majority could give birth when more time is given, (2) it could reduce adverse maternal and newborn outcomes by minimizing delays to receive care, (3) it could reduce unnecessary obstetric bed occupancy in the already overburdened hospitals and allow hospital beds to be used for mothers in critical need, (4) it could also be cost effective to the mother as well as to the health system as hospital care is more costly than care in primary care settings. In such cases, administering a loading dose of prophylactic antibiotic before referral from the health center should be considered to reduce the risk of chorioamnionitis [5, 6]. Nevertheless, in order to implement a policy of expectant management in the primary HCs, hospitals should be able to ensure immediate back up when complications arise. With this regard, lessons can be learned from a case in Zambia that successfully reduces the burden of referrals by strengthening the primary care centers in Lusaka [16].

One of the most striking findings of the study is that, 28 % of the mothers referred to hospital with PROM were not admitted in the hospital they were referred to. Such denials of care at the referral hospitals defies the fundamental notions of having a referral network and delays mothers from accessing appropriate and timely obstetric care and could increase the risk of adverse maternal and perinatal outcomes. In 2011, Addis Ababa recorded highest rate of stillbirth in the country i.e. 30/1000 births where most of these deaths occurred during the intrapartum period [25] and the risks were high among referred cases [26].

More than a third of the term PROM referrals in this study were from the public hospitals. Lack of obstetric bed and unavailability of senior doctors were the two major reasons cited for referring the PROM cases from one public hospital to another. The public hospitals offering comprehensive EmONC in Addis Ababa have one-third less obstetric bed than the World Health Organization (WHO) minimum recommended standard, although the city has sufficient number of hospitals providing comprehensive obstetric care for the population [27]. The primary care facilities offering basic EmONC, by contrast, have more obstetric beds for their caseload. And yet, 30 % of the low risk mothers who visited these basic EmONC centers for intrapartum care were referred to hospitals with the claim that they required advanced care. It should be noted that this proportions does not include antenatal referrals for high-risk mothers. Consistent with our findings, a study in Tanzania reported 24 % complications out of the total deliveries occurring in primary health centers [14], while in Zimbabwe 30 % mothers in primary care settings were referred during antenatal and 13 % during the intrapartum period [13]. The figure in our study is twice the WHO estimate of 15 % obstetric complications that could happen during pregnancy and labour that require referral from basic to comprehensive EmONC facilities [27]. Owing to the high proportions of low risk mothers being referred, about two-thirds of mothers in Addis Ababa are giving birth in the public hospitals [25]. This figure is about four times higher than the expected proportion of hospital deliveries [27]. Moreover, having low rates of caesarean deliveries (only 8 %) in the referral hospitals might also indicate that the majority of the referred cases would not warrant referrals [25]. In a recent study from Addis Ababa, the over-crowded referral hospitals are presenting serious challenges for proper functioning of the referral network [12].

The overwhelming demand for obstetric beds in public hospitals created due to the over-diagnosing and misdiagnosing of obstetric complications in the primary HCs is adding to the problem of bed shortages. One can conclude that the public hospitals in Addis Ababa are busy attending mostly normal labour that could be attended at the primary HCs. According to our findings, the workload could not be a possible reason for the providers to refer large proportions of labouring mothers from the HCs. These health centers have nearly twice more skilled attendants compared to the ratio of skilled providers per attended deliveries, recommended by the United Nations Population Fund in 2011 [23]. A former study also reported that the primary health centers in Addis Ababa has excess skilled attendant for the number of deliveries they mange [11]. Moreover, as shown in Table 3, HCs reporting high rate of referrals and PROM referrals were having a comparable workload with those reporting less referrals.

These failures on the part of the providers to correctly identify obstetric complications that require hospital care and the possibility of making an over-diagnosis could be attributed to; first, to poor knowledge and skills of professionals owing to low quality pre-service training, patchy in-service trainings and lack of continued medical education. Studies reported gaps in in-service training and critical competence on common obstetric and neonatal conditions among providers at the primary HCs in Addis Ababa and elsewhere [11, 12, 14]. A study on midwifery and nursing education in Ethiopia and two other African countries identified serious gaps in the midwifery and nursing curricula to ensure graduates with the required obstetric knowledge and skills [28]. Second, unavailability of management guidelines for most obstetric conditions at the primary care settings could also contribute to the high proportions of misdiagnosis. In this regard, developing clinical guidelines taking into account the local context and realities should be prioritized by concerned stakeholders for improving the quality of obstetric care and outcomes. Developing the guidelines could not be sufficient unless and otherwise it is accompanied by a system that encourage and motivate providers to use them properly and that make them accountable for their action. It was shown that in Zimbabwe failures to adhere to existing clinical recommendations were by large responsible for the high proportions of antenatal and intrapartum referrals from primary care settings [13]. We cannot also exclude fear of accountability among providers as a potential reason for the high rate of referrals from the HCs.

Using a mixed methods approach is one of the strengths of the study that enabled us to make a plausible explanation for the observed high mis-diagnosis and over-diagnosis of PROM. Using a sequential explanatory design has increased our efficiency by making focused interviews. The random selection of the health facilities one from each sub-city could have improved the external validity of the study. However, care should be taken in interpretation of the study findings in terms of availability and accessibility of health facilities, infrastructure, human resource and logistics. Using a retrospective routine data could have been a major weakness of our study if it was not complemented by prospective qualitative interviews.

## Conclusions

This descriptive study complemented with qualitative interviews identified inappropriate referrals to hospitals and explored their possible reasons. Wide variations were observed across the HCs in diagnosis and management approaches for term PROM. The presumably large proportion of obstetric referrals in general and PROM referrals in particular from the HCs appears to be associated with gaps in providers’ competence and the unavailability of practice guidelines. The unavailability of clinical guidelines for most obstetric complications in the primary care might be responsible for the reported variability in diagnosis and management approaches. In-service training for the skilled providers and introducing standard management guidelines for common obstetric complications might improve the quality of obstetric care and outcomes. The study also illustrates alternative approaches in assessing and improving the appropriateness of obstetric referrals, which could be used in similar settings and other contexts, which may be of some usefulness to other health professionals and planners.

## Additional files

10.1186/s13104-016-1852-6 Consolidated Criteria for Reporting Qualitative Research (COREQ) checklist copied from Tong et al. (2007).
10.1186/s13104-016-1852-6 Interview guide used for the in-depth interviews.

## Abbreviations

AACAHB: Addis Ababa City Administration, Health Bureau
EmONC: Emergency Obstetric and Neonatal Care
HC: health center
HCs: health centers
PROM: premature rupture of membranes
WHO: World Health Organization
