# Supplementary material for: High proportions of obstetric referrals in Addis Ababa: the case of term premature rupture of membranes
Source: BMC Res Notes. 2016 Jan 25;9:40. doi: 10.1186/s13104-016-1852-6 (PMC4724955; doi:10.1186/s13104-016-1852-6)
Supplement: Supplementary file 1 — 10.1186/s13104-016-1852-6 Consolidated Criteria for Reporting Qualitative Research (COREQ) checklist copied from Tong et al. (2007). [file 13104_2016_1852_MOESM1_ESM.docx]

Consolidated criteria for reporting qualitative studies (COREQ); 32-items checklist ^[[1]](#footnote-1)^

1. Copied from Tong et al, [↑](#footnote-ref-1)
