# Supplementary material for: High proportions of obstetric referrals in Addis Ababa: the case of term premature rupture of membranes
Source: BMC Res Notes. 2016 Jan 25;9:40. doi: 10.1186/s13104-016-1852-6 (PMC4724955; doi:10.1186/s13104-016-1852-6)
Supplement: Supplementary file 2 — 10.1186/s13104-016-1852-6 Interview guide used for the in-depth interviews. [file 13104_2016_1852_MOESM2_ESM.doc]

**Interview guide to explore skilled providers experiences on diagnosing and managing premature rupture of membranes (PROM)**

**Instruction**:- this guide has three parts, which contains close and open-ended questions.

Date of interview ___________________

Place of interview (health center): ________________________

Interviewer: ________________________

1. **Background information**

- Age ________
- Sex ________
- Professional background a) midwife b) nurse
- How many years of professional training did you have? _______
- How many years of professional experiences do you have in total? _______
- How many years have you been working in the labour ward? ________
- Have you ever received in-service training about PROM? _________

1. **Diagnosing PROM**

- How do you define (premature rupture of membrane) PROM?
- What signs and symptoms do you look for diagnosing PROM?
- For women coming to your clinic with leakage of amniotic fluid what would you do to confirm the leakage?
- What is the procedure that you follow to make PROM diagnosis?
- How long a woman with leakage of amniotic fluid should be monitored to find out labour progress?
- How do you differentiate pre-term from the term PROM?

1. **Managing term PROM**

- How do you manage a woman coming to your clinic with term PROM?
- What kind of guidelines do you follow for managing term PROM in your health facility?
- What are the possible complications that a woman with term PROM should be monitored for?
- How to monitor complications?
- What do you do to prevent possible complications while the woman is in your health facility?
- How long should a woman with term PROM be monitored for labour progress at your health facility before referring her to hospital?
- What are the danger signs for referring a woman with term PROM to hospitals?
- Once you decide on referring a woman with term PROM, please describe me the procedures that you are following and how the referral network works?
